# Harnessing chemically crosslinked microbubble clusters using deep learning for ultrasound contrast imaging

**DOI:** 10.1117/1.JMI.12.4.047001

**Published:** 2025-07-12

**Authors:** Teja Pathour, Ghazal Rastegar, Shashank R. Sirsi, Baowei Fei

**Affiliations:** a University of Texas at Dallas, Department of Bioengineering, Richardson, Texas, United States; b University of Texas at Dallas, Center for Imaging and Surgical Innovation, Richardson, Texas, United States; c UT Southwestern Medical Center, Department of Radiology, Dallas, Texas, United States

**Keywords:** ultrasound, contrast-enhanced ultrasound imaging, machine learning, deep learning, anomaly detection, clustered microbubbles, contrast agents

## Abstract

**Purpose:**

We aim to investigate and isolate the distinctive acoustic properties generated by chemically crosslinked microbubble clusters (CCMCs) using machine learning (ML) techniques, specifically using an anomaly detection model based on autoencoders.

**Approach:**

CCMCs were synthesized via copper-free click chemistry and subjected to acoustic analysis using a clinical transducer. Radiofrequency data were acquired, processed, and organized into training and testing datasets for the ML models. We trained an anomaly detection model with the nonclustered microbubbles (MBs) and tested the model on the CCMCs to isolate the unique acoustics. We also had a separate set of control experiments that was performed to validate the anomaly detection model.

**Results:**

The anomaly detection model successfully identified frames exhibiting unique acoustic signatures associated with CCMCs. Frequency domain analysis further confirmed that these frames displayed higher amplitude and energy, suggesting the occurrence of potential coalescence events. The specificity of the model was validated through control experiments, in which both groups contained only individual MBs without clustering. As anticipated, no anomalies were detected in this control dataset, reinforcing the model’s ability to distinguish clustered MBs from nonclustered ones.

**Conclusions:**

We highlight the feasibility of detecting and distinguishing the unique acoustic characteristics of CCMCs, thereby improving the detectability and localization of contrast agents in ultrasound imaging. The elevated acoustic amplitudes produced by CCMCs offer potential advantages for more effective contrast agent detection, which is particularly valuable in super-resolution ultrasound imaging. Both the contrast agent and the ML-based analysis approach hold promise for a wide range of applications.

## 1 Introduction

Contrast-enhanced ultrasound (CEUS) imaging is a cost-effective and portable imaging modality that combines ultrasound imaging with microbubble-based ultrasound contrast agents (UCAs). These microbubbles (MBs), encapsulated in shells of lipid, protein, or polymer,^1^ act as vascular contrast agents circulating in the blood pool. With a compressible gas core, UCAs efficiently scatter ultrasound energy, inducing volumetric oscillations in response to an ultrasound field. This dynamic behavior enhances ultrasound image contrast.^2^^–^^5^ The unique acoustic characteristics of scattered echoes from MBs can be distinguished from surrounding tissues using harmonic and subharmonic frequency response,^6^^,^^7^ or nonlinear pulsing sequences,^8^ thereby improving the signal-to-noise ratio (SNR) and overall image contrast.^6^^,^^9^^–^^11^

Recent advancements in beamforming and image processing techniques have aimed to enhance both lateral and axial resolution of ultrasound imaging.^12^^–^^14^ Notably, the utilization of contrast agents has demonstrated the potential to surpass the conventional ultrasound resolution limit, achieving resolutions lower than the limit through super-resolution ultrasound (SR US) imaging.^15^^–^^17^ The application of deep learning algorithms to SR US imaging has further contributed to resolution improvement.^18^^,^^19^ Despite these advancements, there is a notable lack of research focusing on the development of novel contrast agents to enhance ultrasound imaging modalities. Some studies have shown that there are residual harmonics and subharmonic signals generated from the tissues that may result in reduced SNR.^20^^–^^22^ We need better contrast agents that can be localized and detected with high SNR and accuracy. Studies indicate that novel contrast agents could enhance contrast agent detection accuracies and SNR, thereby improving lateral resolution.^17^^,^^23^^,^^24^ Therefore, the development of better contrast agents is essential, which are both localized and detectable.

Recently, researchers have delved into the effects of multiple MBs in close proximity. This phenomenon can occur in the form of MB clouds^25^ or as a result of ultrasound-induced clustering.^26^^,^^27^ The concept of MB clusters has garnered attention in the literature, suggesting that interactions among MBs may lead to an enhanced response or yield unique acoustic signatures that differ from those produced by individual contrast agents spaced apart. Wang^25^ conducted a study examining the impact of MB sizes on the collapse of MB clouds and the resulting acoustics from bubbles of varying sizes within the cloud. Significantly, the acoustic emissions and collapse phenomena were found to be highly dependent on the distribution of MB sizes within the cloud. Another compelling piece of evidence supporting the notion that MB clusters may offer distinctive acoustic properties for imaging applications comes from Omta’s^28^ work that investigated the interaction of multiple MBs under low and high acoustic pressures. The study revealed that the frequency spectrum of the emitted sound exhibited a peak at a frequency much lower than the eigenfrequency of individual bubbles, suggesting that MB clustering could yield unique acoustic responses beneficial for improved *in vivo* detection. Several research groups have demonstrated the capability of MBs to aggregate under ultrasound exposure.^29^^–^^32^ Their research highlighted that the clustering phenomenon can significantly influence the acoustic response, emphasizing its crucial importance in biomedical applications.^26^

Our group recently introduced a novel type of UCA named “chemically crosslinked microbubble clusters” (CCMCs).^33^ These clusters, composed of lipid MBs, are chemically crosslinked through an [azide:dibenzocyclooctyne (DBCO)] copper-free click chemistry approach^34^ and consist of peripheral MBs surrounding a core MB. The aim of this study was to employ machine learning (ML) techniques to investigate if CCMCs exhibit unique acoustic signatures that can be detected using an ultrasound scanner and machine learning. Expanding on our previous research,^35^ where we demonstrated the distinctive acoustic properties of CCMCs using a basic artificial neural network. Our previous research focused on the detection of CCMCs in large populations of bubbles. Here, we reduced the concentration of MBs to isolate individual occurrences and applied a novel anomaly detection model for effective classification and separation of clusters in the CCMC bubble population. Although traditional signal processing methods such as spectral analysis provide useful insights into the acoustic properties of MB-based contrast agents, these methods can fall short when analyzing subtle, variable, and overlapping features—particularly at low concentrations. In such cases, distinguishing clustered MB signatures from individual UCAs can be ambiguous using fixed thresholding approaches. Therefore, we employed a deep learning–based anomaly detection model, which is well suited for capturing complex and nonlinear patterns in high-dimensional data and does not rely on predefined signal features.

Overall, we developed a reliable approach for the classification of CCMC acoustics. Upon closer examination, leveraging an anomaly detection model based on autoencoders,^36^^–^^39^ we successfully isolated the unique acoustics originating from CCMCs. Notably, these isolated acoustics exhibited higher amplitude power in both the fundamental and subharmonic regions when compared with the control groups. Our comprehensive approach enabled the isolation of individual microbubble events and the distinct acoustics associated with CCMCs.

## 2 Materials and Methods

### 2.1 Preparation of CCMCs and Individual UCAs

**Fig. 1:**
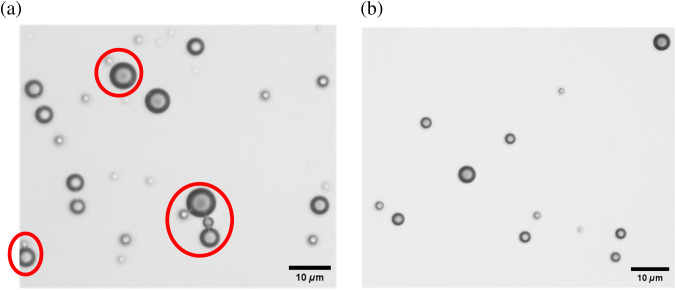
Microscopy images of CCMCs and negative control samples. (a) Clustering of microbubbles is observed and highlighted in a red circular region. (b) No clusters are observed in the negative control samples.

CCMCs are synthesized using a methodology similar to the approach described by Hall et al.,^33^ employing copper-free click chemistry. In brief, two distinct UCA samples were prepared, one containing DSPC and DSPE-PEG5K-DBCO, whereas the second sample consisted of DSPC and DSPE-PEG5K-Azide, following the detailed procedure delineated by Sirsi et al.^40^ These UCAs are subsequently mixed in a fixed concentration ratio of 1:10 (DBCO:azide) and subjected to a 1 h incubation at 4°C. As part of the negative control, sodium azide was used to block the DBCO–azide chemistry, thereby preventing the formation of bubble crosslinks. Prior to data collection, all samples underwent validation through examination under a brightfield BX50 Upright Microscope equipped with an ACH 60×/0.80 ∞/0.17 objective. Notably, the chemical crosslinking imparts a cluster formation observed in the sample of CCMCs, as depicted in Fig. 1(a), highlighted in a red circular region, whereas the negative control, represented in Fig. 1(b), reveals the presence of individual UCAs when observed under the microscope. It is important to note that clusters are seen in almost 25% of the overall CCMC UCA sample.^33^

### 2.2 Data Acquisition

Following the validation of the samples for the presence of clusters and individual UCAs, radiofrequency (RF) data were systematically acquired using a GEM5ScD clinical phased array transducer and the Verasonics Vantage 256 system (Redmond, Washington, United States).

**Fig. 2:**
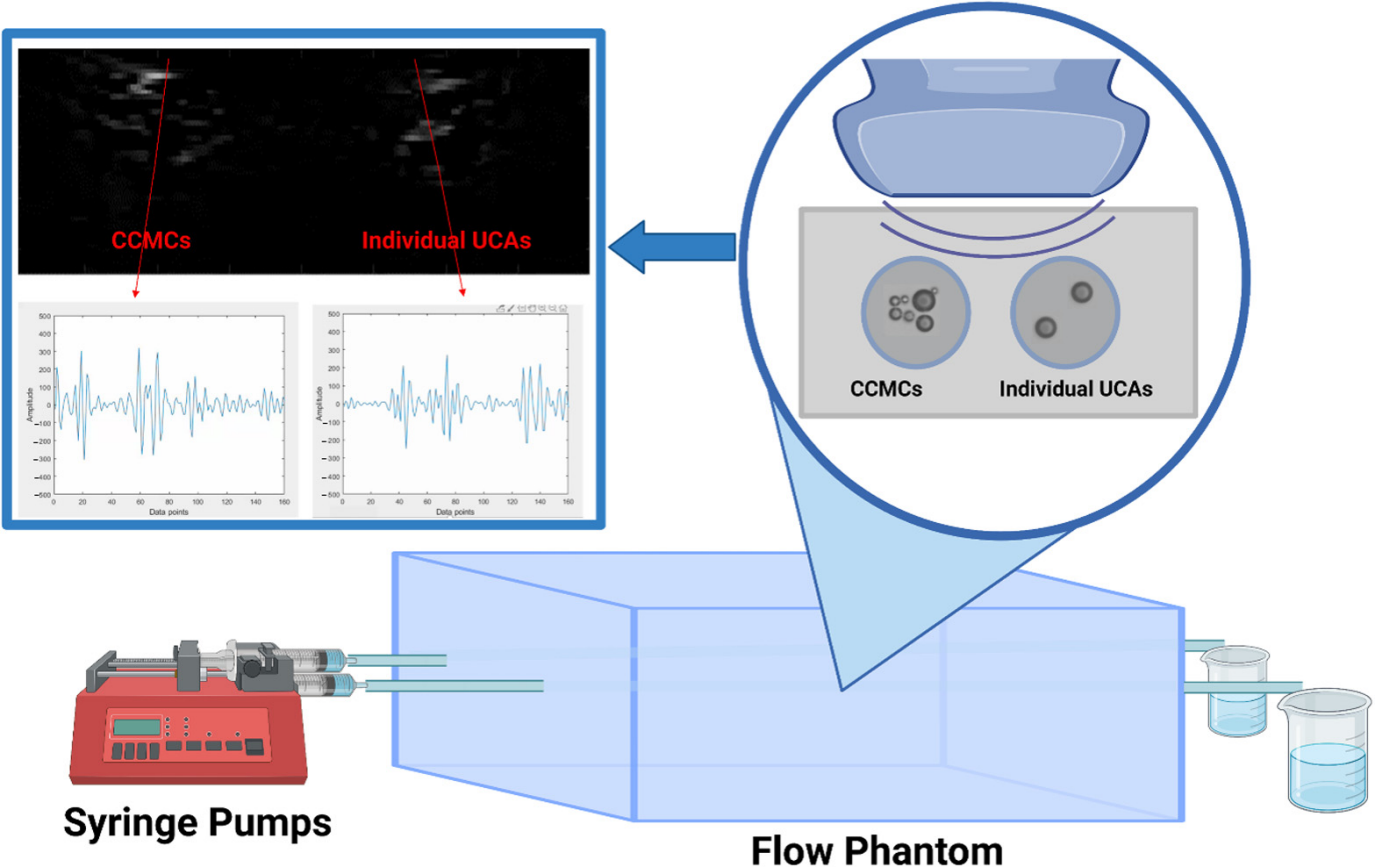
Description of the experimental setup employed for RF data and B-mode image acquisitions. The syringe pump enables the simultaneous injection of the UCA solution, containing CCMCs and individual UCAs, into the two tunnels of the flow phantom, and the data are acquired using a clinical probe.

Figure 2 illustrates the setup with a flow phantom, featuring two parallel tunnels, each transporting CCMCs and the negative control, respectively. These parallel tunnels are simultaneously exposed to ultrasound, and the RF data from both tunnels are captured within a single frame. This concurrent data collection for CCMCs and the negative control serves to mitigate any potential data variability that may arise from sequential dataset acquisition.

We employed a low-concentration method to isolate individual UCA events, utilizing a total of 10 UCAs per milliliter. This concentration was determined using a Multisizer 4e Coulter Counter, Beckman Coulter (Brea, California, United States), followed by dilution with phosphate-buffered saline. Both CCMCs and individual UCA samples were prepared using this concentration. In addition, a fixed frame rate of 100 frames per second was utilized throughout the experiments. A 20 mL syringe, connected to a syringe pump operating at a constant flow rate of 5 mL/min, was employed to infuse the solution through the phantom.

### 2.3 Data Processing

The ultrasound data (RF data and B-mode images) obtained from both sample sets undergo a sequence of data processing procedures, as described below. Here, it should be noted that “frames” refer to individual instances of captured ultrasound data, whereas “dataset” denotes a collection of these frames used for analysis.

**Fig. 3:**
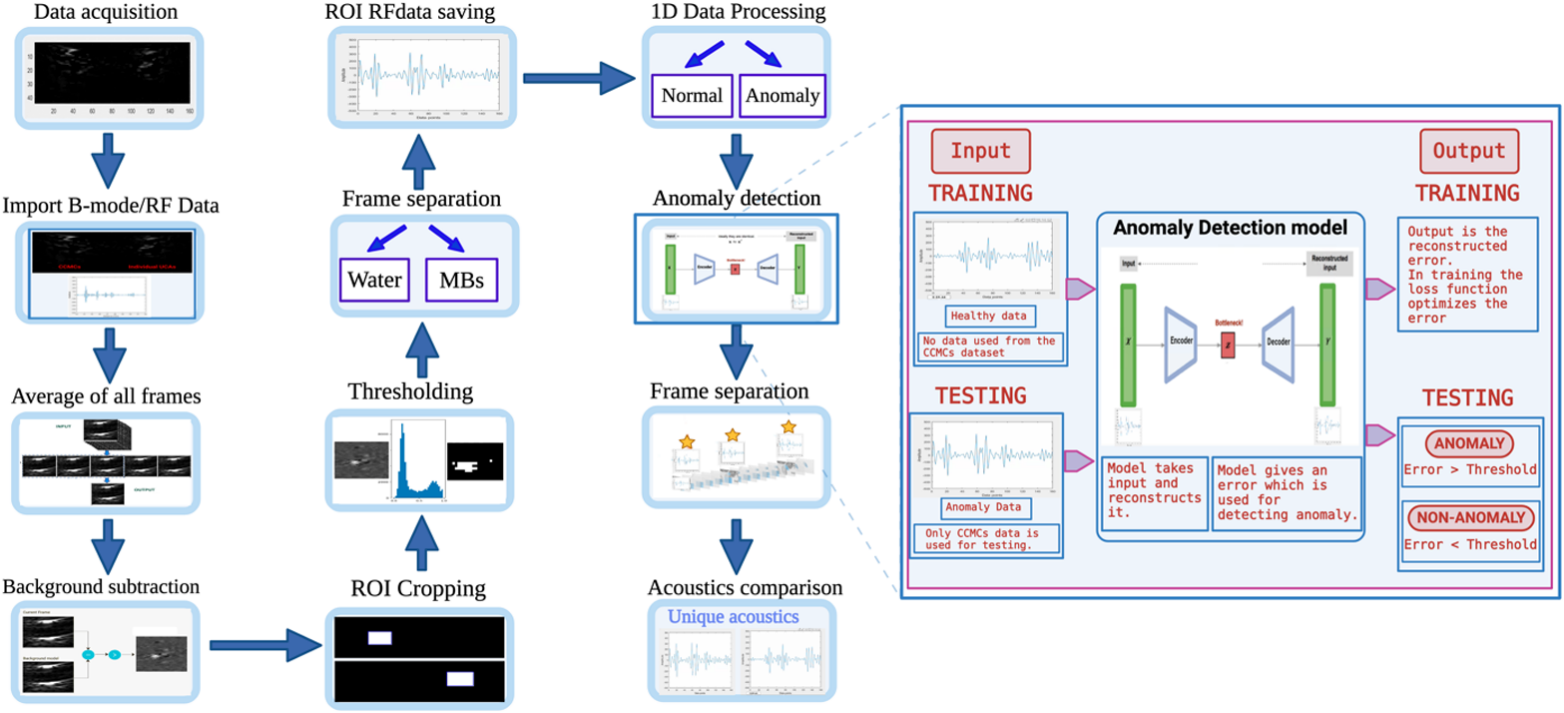
Illustration of a comprehensive workflow outlining step-by-step data processing and analysis procedures. The magnified image offers insight into the functionality of the anomaly detection model. Subsequent analysis involves the separation and examination of frames to compare the acoustics associated with the detected anomalies.

1. Averaging and background subtraction
  The mean of all the frames is computed and subtracted from all the frames individually. This procedure eliminates reflections from the tunnel walls and other unwanted noise within the images, thereby emphasizing the signals originating from UCAs. This enhancement simplifies the process of isolating and distinguishing these signals.
2. Cropping the region of interest
  The subtracted B-mode images undergo cropping to delineate the region of interest (ROI), which corresponds to a rectangular section within the tunnel containing UCAs, as illustrated in Fig. 3. The selection of the ROI is performed manually for the datasets as the precise location of the ROI may fluctuate during data collection on different days.
3. Thresholding to highlight UCAs
  A threshold is employed on the subtracted and cropped B-mode images to effectively filter the signals generated by UCAs. This filtering step retains UCA signals while discarding all other signals. Consequently, frames containing UCAs are represented by nonzero pixel values, whereas frames without UCAs are indicated by zero pixel values.
4. Separating frames with UCAs
  It should be noted that, due to the low concentrations, a subset of frames lacked the presence of UCAs. To differentiate frames containing UCAs from those where UCAs are absent, B-mode images were used. After thresholding, frames lacking UCAs are systematically eliminated through the implementation of a pixel filter that effectively screens out frames bearing zero pixel values. The filter, set with a threshold requiring a minimum of three pixels to possess nonzero values, consequently, discards frames featuring fewer than three such nonzero pixels. This process effectively segregates the frames containing UCAs.
5. Generating datasets for the ML algorithm
  Once the frames containing UCAs were isolated, we proceeded to assemble datasets tailored for anomaly detection models. We followed the approach described in Pathour et al.,^41^ employing 1D RF datasets. Specifically, the RF data corresponding to the frames with UCAs, after their separation, were backtracked using the B-mode images. For each sample set, we focused on a single element of the transducer located at the center of the respective tunnels. The 1D RF data extracted from the center of the tunnel within the cropped ROI were preserved as the representative samples and were saved as either CCMCs or individual UCA data.

All the steps (1 to 5) were performed in MATLAB for both sample sets (CCMCs and individual UCAs), as shown in Fig. 3.

### 2.4 Training and Testing Datasets for Anomaly Detection Model

The 1D RF data underwent processing and were then organized into CCMCs and individual UCAs, respectively. The training dataset consisted of the acoustic response of individual UCA, whereas CCMC data were not included. The testing dataset comprised exclusively of CCMC data as it contained acoustic responses from clusters within the CCMC sample. The training and testing datasets comprised 45,960 and 16.519 frames, respectively, each containing 160 amplitude values.

### 2.5 Anomaly Detection Model

We employed an anomaly detection algorithm to identify the distinctive acoustic response of CCMCs. Specifically, we utilized an autoencoder-based anomaly detection model,^38^ consisting of an encoder, a decoder, and a bottleneck layer. The encoder network comprised three long short-term memory (LSTM) layers with progressively decreasing kernel sizes, and the decoder network mirrored this structure with three LSTM layers featuring increasing kernel sizes.^42^ Sigmoid activation function^43^ was applied in conjunction with the Adam optimizer^44^ and the mean squared error loss function.^45^ The anomaly detection model architecture, comprising LSTM-based encoder and decoder layers, which was adapted from previous frameworks, applied to general sensor anomaly detection tasks. However, in this study, it was specifically tailored to process the temporal dynamics inherent in one-dimensional ultrasound RF signals. These signals exhibit sequential patterns influenced by the acoustic behavior of contrast agents. By training the model exclusively on individual UCA signals, it learns a baseline representation of normal acoustic behavior. Deviations from this learned representation, as seen with CCMCs, are flagged as anomalies.

**Fig. 4:**
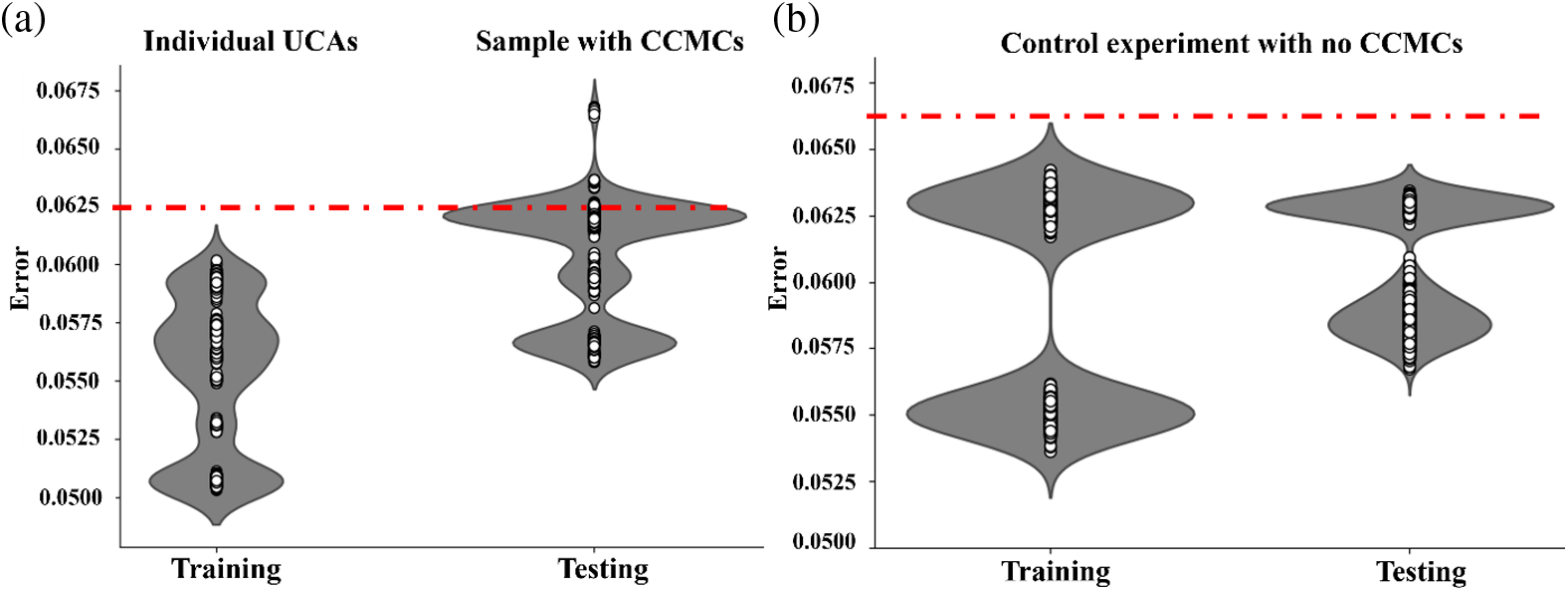
Error values generated by the anomaly detection model are depicted in (a). The error violin plot illustrates the training dataset without CCMCs and the testing dataset with CCMCs, with the threshold determined based on training error values. (b) Error values from the control experiment, following the same procedure as in panel (a), revealing that the error values do not surpass the established threshold.

In this autoencoder model, the encoder layer learned the acoustic response of individual UCAs while compressing the data into a lower dimensional representation, and the decoder layer aimed to reconstruct the input to closely match the original input. The reconstruction error, calculated as the difference between the input and the reconstructed output, serves as a measure of how well the model captures the input’s features. In this study, we regarded the acoustic response of individual UCAs as the “normal dataset,” whereas CCMCs were categorized as the “anomaly dataset.” During the training phase, the model learned the variations in the acoustic signals of conventional UCAs using the normal dataset. During testing, we employed an anomaly dataset, which contained acoustic responses from clusters. Roughly 25% of the UCAs sample consisted of CCMCs, with the remaining portion comprising regular lipid MBs. In the testing phase, when the autoencoder model encountered a frame with individual UCA data, the reconstruction error would be low as the acoustic response would be similar to the individual UCAs from the normal dataset. Conversely, when the model encountered a frame with clusters in the sample, the reconstruction error would be high due to the dissimilarity in acoustic response compared with the healthy dataset. We set a threshold for the reconstruction error by analyzing its trends, leveraging the distribution of reconstruction errors from the training phase (Fig. 4). Our approach involved selecting a threshold value exceeding the maximum reconstruction error observed during training, and any frame with a reconstruction error exceeding this threshold will be classified as an anomaly.^46^ This method ensures that the model is highly sensitive to outliers while maintaining specificity as any frame with a reconstruction error greater than what was encountered during training is considered truly anomalous.

### 2.6 UCA Separation Algorithm and Acoustics Comparison

We separated the anomaly frames from the testing dataset. Frames identified as anomalies by the model were stored as anomaly frames, whereas the remaining frames were saved as nonanomaly frames. Frames originating from the healthy dataset are designated as healthy frames. To explore the disparities among these frames, we conducted a comparative analysis of their acoustic responses. We employed frequency domain plots for this purpose. Specifically, we focused on the ROI within the subharmonic and fundamental frequency regions to assess variations among the frames. We created scatter plots depicting the subharmonic versus fundamental frequencies of the ROI, aiming to assess linearity and the degree of separation among the frames.

### 2.7 Control Experiment

We performed a control experiment in which both sample sets contained conventional lipid UCAs instead of CCMCs and individual UCAs. The data acquisition and processing procedures mirrored those of the actual experiment. In this control experiment, we expected that none of the frames would be categorized as anomalies by the model. In addition, we conducted a comparative acoustic analysis among the frames, following the same approach outlined earlier, to determine any differences.

## 3 Results

### 3.1 Anomaly Detection Model Performance

The anomaly detection model utilized the training dataset to assimilate the acoustic signature of individual UCAs by reconstructing the input. Employing a process of dimensionality reduction followed by expansion to the original input size, the model acquired a nuanced understanding of the data’s intricacies and distinctive signature. Demonstrating effectiveness, the model achieved a notably low reconstruction error of 0.0625 [see Fig. 4(a)]. This reconstruction error served as the basis for establishing a threshold, with the maximum error determining the criterion for the testing dataset. Subsequently, the testing dataset underwent assessment using the trained anomaly detection model. Similar to the training phase, the model sought to reconstruct the input, generating a reconstruction error. Instances, where the error exceeded the threshold, indicated a unique acoustic signature, distinct from that of individual UCAs, suggesting distinctive acoustic characteristics associated with clusters. Frames displaying errors surpassing the threshold were identified and categorized as anomalies [see Fig. 4(a)].

### 3.2 Validation Results from Control Experiments

The control experiment indicated that the model effectively identified anomalies. Both the training and testing datasets underwent training utilizing the same anomaly detection model. Consistent with the actual experiment, the threshold was established using the reconstruction error from the training phase. During the testing phase, frames exhibiting a reconstruction error surpassing the threshold were designated as anomalies. As anticipated, no frames with reconstruction errors higher than the threshold were observed during the testing phase [Fig. 4(b)]. This absence of distinct acoustics in the control experiment, compared with the actual experiment, suggests a similarity in the acoustic responses of individual UCAs. The purpose of this experiment was to demonstrate that the anomaly detection model can proficiently learn and reconstruct inputs with low error for datasets containing individual UCAs while encountering higher reconstruction errors for datasets with clusters due to their unique acoustic responses.

### 3.3 Analysis and Comparison of Anomalous Frames

**Fig. 5:**
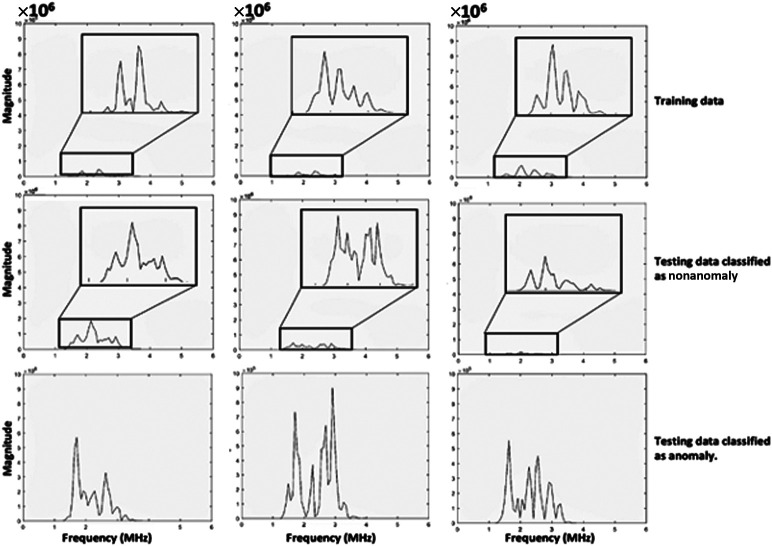
Raw frequency plots are presented for the training data (individual UCAs), testing data classified as nonanomalies, and testing data classified as anomalies.

The dataset was divided into frames classified as anomalies, which were then compared with the training, nonanomaly, and control datasets. The nonanomaly dataset comprised frames with reconstruction errors below a specified threshold. To assess the differences among these groups, frequency domain plots were analyzed. Figure 5 displays the frequency plots of individual frames from each group. Notably, the frequency plots of anomaly frames within the testing dataset revealed a higher area under the curve and peaks in the subharmonic and fundamental regions. Furthermore, the fundamental and subharmonic peaks for the training and nonanomaly groups in the testing dataset exhibited a comparable range, in contrast to those of the anomaly group.

**Fig. 6:**
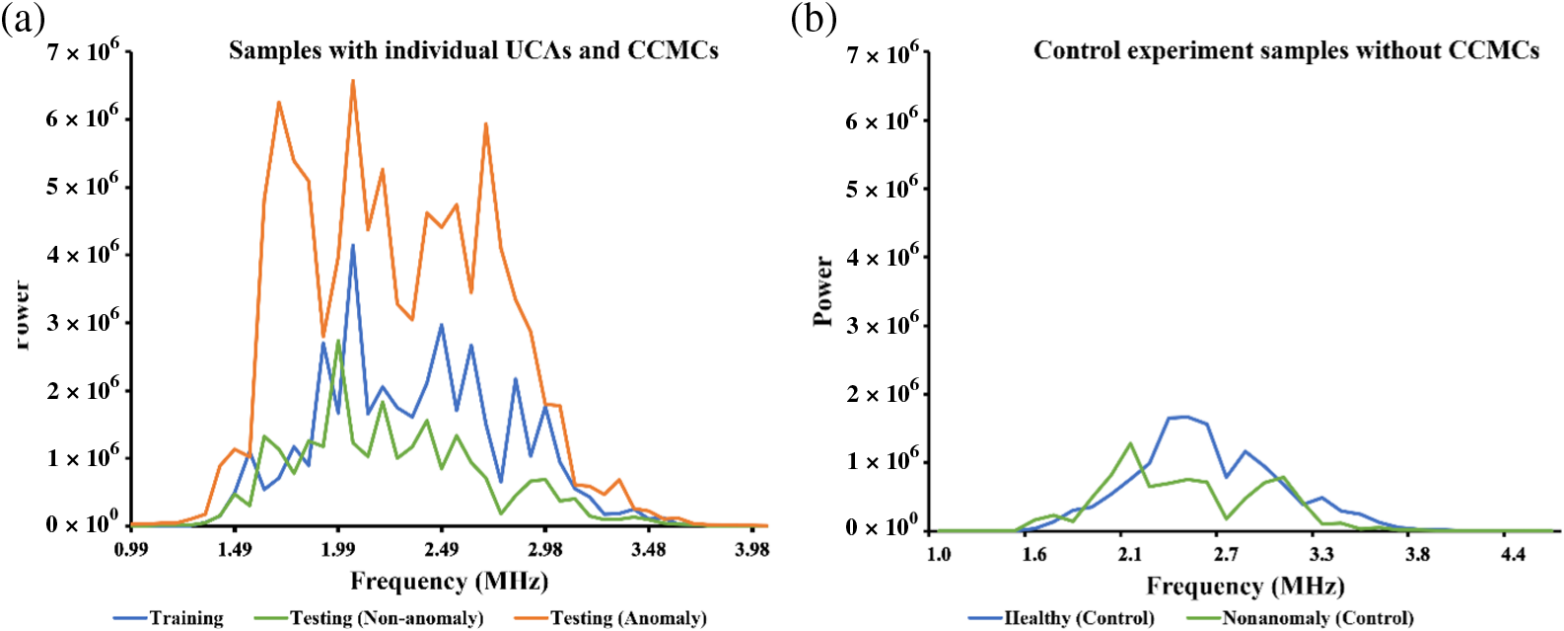
Comparison of mean frequencies in training and testing datasets is illustrated in panel (a). This includes a comparison between the training datasets (individual UCAs) and testing datasets with and without anomalies. (b) Specifically focuses on the frequency comparison of control experiments, involving both training and testing datasets without anomalies.

Furthermore, a comparison was made using the mean frequency plots of all frames across the three groups, presented in Fig. 6. It is evident that the anomaly group within the testing dataset exhibited higher peaks and areas under the curve compared with the other groups [Fig. 6(a)]. To provide a broader context, a graph from the control dataset was included [Fig. 6(b)], illustrating that the frequency peaks align with the remaining groups, except for the anomaly group. The collective findings suggest that anomaly frames within the testing dataset display elevated power and energy compared with the other datasets. This observation is consistently supported by both the frequency plots of individual frames and the mean frequency plots across all groups.

### 3.4 Scatter Plot Comparison: Fundamental versus Subharmonic

**Fig. 7:**
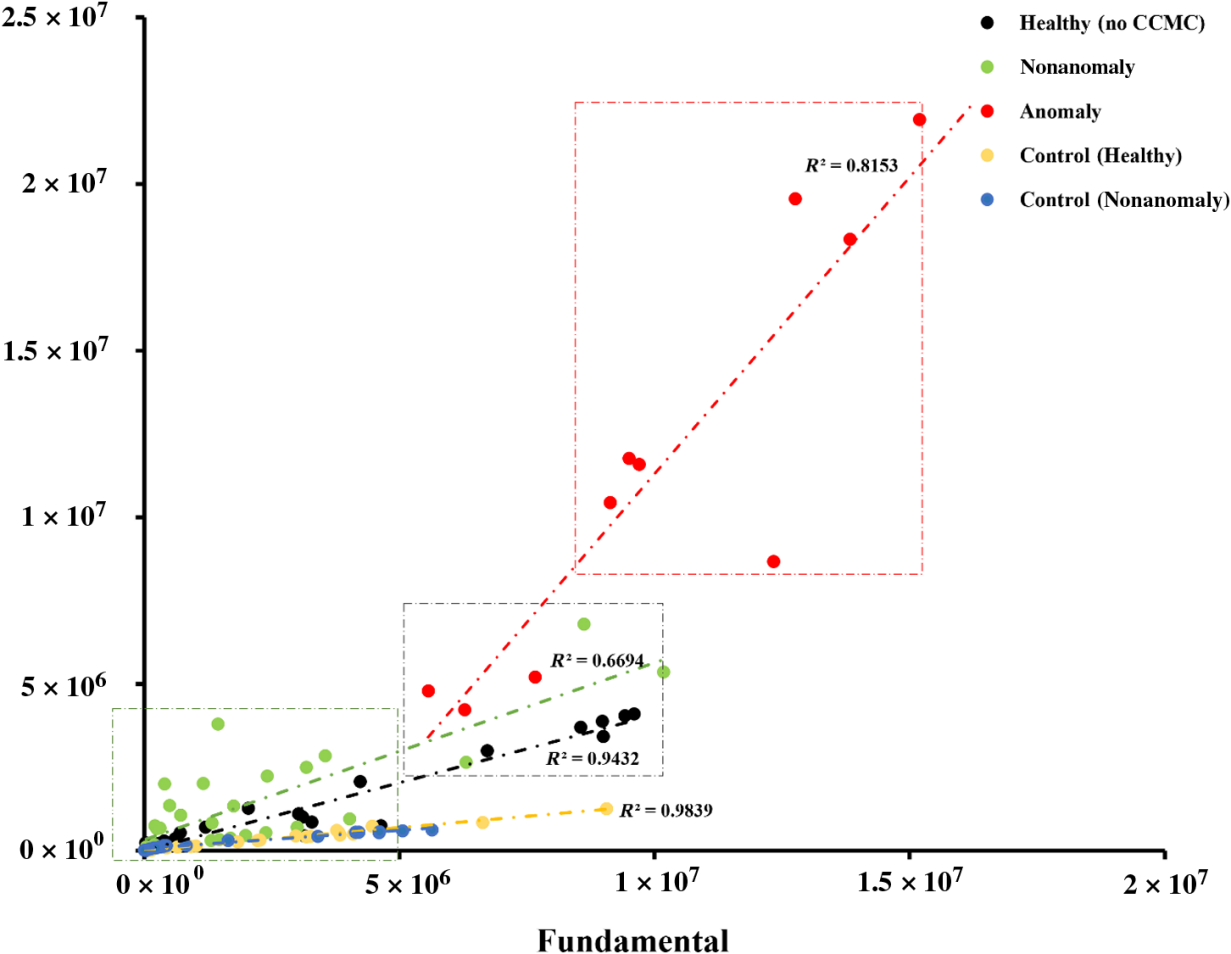
Scatter plot comparing mean subharmonic versus fundamental amplitude values for training data (individual UCAs), testing data without anomalies, testing data with anomalies, and control experiment datasets. Anomalies, exhibiting higher power, are highlighted in a rectangular box, whereas the remaining data are grouped in a green box denoting lower power. Linear regression lines with corresponding r-squared values are shown and indicate a higher slope for the anomaly dataset.

We selected a region of interest in the fundamental and the subharmonic region in the frequency domain analysis, and they were plotted in a scatter plot to compare the amplitudes at the fundamental and the subharmonic frequencies for all the data groups, as shown in Fig. 7. We observed that the scatter points of anomalies from the testing group had a higher ratio, which suggests higher energy and could be clustered into one. No other data group was clustered into this group (Fig. 7). The rest of the groups had lower ratios suggesting low energy and were clustered into another group. There were a few scatter points in the middle that could not be classified into either of the groups. This might be due to the larger bubbles in the individual UCA’s solution that was used in the training datasets and due to small clusters, which might have generated lower power compared with the other clusters.

## 4 Discussion

In this study, we developed a machine learning algorithm that detects and isolates unique acoustics of CCMCs using one-dimensional RF data from a clinical transducer, and we propose that this novel ultrasound imaging technique could lead to new methods of detecting contrast agents *in vivo* with greater accuracy and sensitivity. Previously, we had shown that the acoustics of CCMCs are distinctive when compared with the nonclustered lipid UCAs.^35^ Here, we investigated the distinctive characteristics of individual acoustic events produced by CCMCs using diluted samples. After classification, we used an anomaly detection model based on autoencoders to separate the acoustics that were responsible for the distinction in CCMCs.

In the scatter plot depicted in Fig. 7, it is evident that anomalies from the CCMCs dataset (red rectangular region) exhibit notably higher amplitude and energy in frequency domain analysis compared with the remainder of the dataset, including the control group. We believe that this might be due to the coalescence event of CCMCs exposed to a low-intensity ultrasound pulse, as shown by Hall et al.^33^ Postema et al.^47^ studied the coalescence events of MBs under ultrasound. During the coalescence event, two bubbles approach collision, potentially leading to the flattening of the adjacent bubble surface before contact. The liquid trapped among the bubble walls then begins to drain into the surrounding medium, reaching a critical thickness where the film becomes unstable, resulting in rupture and formation of a merged bubble. This phenomenon involves the rapid expulsion of liquid from the adjacent bubbles, which we hypothesize results in the generation of unique acoustics. However, it is important to note that the coalescence of bubbles was not directly observed in this study.

Another interesting observation evident from Fig. 7 is the presence of scatter points in the gray rectangular region. These scatter points exhibit fundamental to subharmonic frequency ratios higher than the green rectangular region and lower than the red rectangular region. We believe that this could be due to the larger bubbles present (from crosslinking or coalescence), potentially leading to higher amplitude and energy generation. This observation aligns with the findings of Morgan et al.^48^ Although conventional spectral analysis was used to support our findings and interpret the model’s output, it alone was not sufficient to reliably distinguish clustered CCMCs from individual UCAs—particularly due to overlapping frequency components that limited the specificity of threshold-based methods. By contrast, our machine learning–based approach, specifically the autoencoder anomaly detection model, demonstrated the ability to isolate frames with unique acoustic patterns that aligned with the expected higher energy signatures of CCMCs. This model captures complex, nonlinear relationships within the RF data without relying on manually defined thresholds or handcrafted features, offering greater robustness to signal variability and subtle differences in microbubble behavior. Furthermore, its scalability and adaptability to real-time or streaming data make it highly suitable for high throughput and clinical applications. Although we were unable to report standard detection metrics such as accuracy or sensitivity due to the absence of frame-level ground truth labels, we evaluated model performance using a combination of approaches. These included the separation of reconstruction error distributions among experimental and control datasets, frequency domain analyses of detected anomalies, and a control experiment validating model specificity. Together, these results confirm the model’s ability to isolate frames with distinct acoustic patterns characteristic of CCMCs. The distinct acoustic properties of CCMCs, combined with the strength of this deep learning approach, suggest promising potential in CEUS and super-resolution imaging—particularly in clinical scenarios that demand high resolution and high SNR, such as vascular imaging, tumor localization, and the monitoring of targeted therapies. In such settings, CCMCs could serve as an effective contrast agent to improve detection accuracy and diagnostic confidence.

## 5 Study Limitations

Although this study presents valuable insights, it is important to acknowledge certain limitations. First, we were unable to access ground truth data and capture the acoustics of CCMCs during the coalescence event as it was beyond the scope of this study. However, these limitations provide opportunities for future research endeavors. Moreover, we could not conclusively verify whether the observed higher amplitudes and energy generated from CCMCs were solely attributable to the coalescence event. In addition, it is noteworthy that the yield of cluster formation is ∼25%, indicating potential room for improvement in manufacturing strategies to enhance the cluster formation percentage. In future studies, we plan to extend our investigation to include the collection of ground truth data using ultrafast microscopy imaging of CCMCs under ultrasound while simultaneously recording the backscatter signals. This approach holds promise for uncovering further insights and refining our understanding of CCMCs’ behavior under ultrasound conditions. Although this study was limited to phantom-based experiments, we acknowledge the importance of *in vivo* validation. We have not yet performed *in vivo* studies; however, we plan to pursue them in future work to assess the behavior and imaging performance of CCMCs in physiologically relevant conditions.

Although the autoencoder was trained on a dataset of over 45,000 RF frames providing a broad representation of individual UCA acoustic signatures, we believe that a systematic evaluation of model performance with varying training dataset sizes was not conducted. Such an analysis could help assess the model’s generalizability and robustness, particularly in scenarios with limited training data. Future work will include experiments to evaluate the sensitivity of the model to training dataset size.

## 6 Conclusion

Overall, our study highlights the distinctive acoustic properties exhibited by CCMCs, characterized by heightened amplitudes and energy. These characteristics present promising opportunities for accurate event detection and localization, ultimately contributing to the enhancement of image quality and lateral resolution in CEUS imaging and super-resolution ultrasound imaging.

## Data Availability

The datasets generated and analyzed during the current study are not publicly available.
